# An *Aspergillus nidulans* β-mannanase with high transglycosylation capacity revealed through comparative studies within glycosidase family 5

**DOI:** 10.1007/s00253-014-5871-8

**Published:** 2014-06-21

**Authors:** Anna Rosengren, Sumitha K. Reddy, Johan Svantesson Sjöberg, Oskar Aurelius, Derek T. Logan, Katarína Kolenová, Henrik Stålbrand

**Affiliations:** 1 Department of Biochemistry and Structural Biology, Lund University, P.O. Box 124, 221 00 Lund, Sweden; 2 Present Address: Institute of Chemistry, Center for Glycomics, Slovak Academy of Sciences, Dúbravská cesta 9, 845 38 Bratislava, Slovakia

**Keywords:** β-mannanase, GH5, Transglycosylation, H_2_^18^O, Acceptor, MALDI-TOF MS

## Abstract

β-Mannanases are involved in the conversion and modification of mannan-based saccharides. Using a retaining mechanism, they can, in addition to hydrolysis, also potentially perform transglycosylation reactions, synthesizing new glyco-conjugates. Transglycosylation has been reported for β-mannanases in GH5 and GH113. However, although they share the same fold and catalytic mechanism, there may be differences in the enzymes’ ability to perform transglycosylation. Three GH5 β-mannanases from *Aspergillus nidulans*, AnMan5A, AnMan5B and AnMan5C, which belong to subfamily GH5_7 were studied. Comparative studies, including the GH5_7 TrMan5A from *Trichoderma reesei*, showed some differences between the enzymes. All the enzymes could perform transglycosylation but AnMan5B stood out in generating comparably higher amounts of transglycosylation products when incubated with manno-oligosaccharides. In addition, AnMan5B did not use alcohols as acceptor, which was also different compared to the other three β-mannanases. In order to map the preferred binding of manno-oligosaccharides, incubations were performed in H_2_^18^O. AnMan5B in contrary to the other enzymes did not generate any ^18^O-labelled products. This further supported the idea that AnMan5B potentially prefers to use saccharides as acceptor instead of water. A homology model of AnMan5B showed a non-conserved Trp located in subsite +2, not present in the other studied enzymes. Strong aglycone binding seems to be important for transglycosylation with saccharides. Depending on the application, it is important to select the right enzyme.

**Electronic supplementary material:**

The online version of this article (doi:10.1007/s00253-014-5871-8) contains supplementary material, which is available to authorized users.

## Introduction

β-Mannosidic linkages are abundant in nature, notably present in hemicellulose such as spruce galactoglucomannan and in plant storage galactomannans (Scheller and Ulvskov 2010). These β-mannan polysaccharides are important renewable resources and their enzymatic conversion is of great interest in sustainable biorefinery processes (Carvalheiro et al. 2008). β-Mannanases are the main endo-acting enzymes that hydrolyze β-1,4-linkages in β-mannans (Gilbert et al. 2008). So far, β-mannanases have been classified into glycoside hydrolase (GH) families 5, 26 and 113, as displayed in the CAZy database; www.cazy.org, (Cantarel et al. 2009; Lombard et al. 2014). GH5 is a diverse family, encompassing not only β-mannanases but also a range of other enzymatic activities from a variety of organisms. GH5 enzymes are (β/α)_8_ TIM-barrels and several β-mannanase 3D structures from the family have been published, six of which are from eukaryotic organisms (Bourgault et al. 2005; Couturier et al. 2013; Goncalves et al. 2012; Larsson et al. 2006; Mizutani et al. 2012; Sabini et al. 2000). Although having the same basic catalytic specificity (i.e. hydrolysis of β-mannosidic bonds), there may be differences in the biological functions of β-mannanases; e.g. microbial β-mannanases of saphrophytic fungi take part in the degradation of woody plant tissues (Coutinho et al. 2009) while some plant β-mannanases are involved in germination (Iglesias-Fernandez et al. 2011). Functional differences are also present among bacterial β-mannanases (Tailford et al. 2009). Choosing the right enzyme for a certain biotechnological application, such as for biomass saccharification or for enzymatic synthesis, is crucial. Therefore, comparative biochemical studies of β-mannanases are of great importance to expand knowledge on functional diversity and on structural features that influence specific functions. This will also contribute to an increased understanding of the biological role of β-mannanases. Recently, enzymes in GH5 were classified into subfamilies based on sequence similarities and phylogenetic analysis, which grouped β-mannanases into subfamilies 7, 8, 10 and 17 (GH5_7, GH5_8, GH5_10 and GH5_17) (Aspeborg et al. 2012). Although enzymes with high sequence identity are likely to share similar functions, there might still be differences within subfamilies. However, this has not yet been well studied. β-Mannanases use the retaining double displacement mechanism and can in principle carry out transglycosylation, i.e. enzymatic synthesis of β-mannosidic bonds—arguably the most difficult glycosidic bond to synthesize chemically (Gridley and Osborn 2000). Enzymatic synthesis of glyco-conjugates such as alkyl-glycosides can in general be carried out with transglycosylating glycoside hydrolases which use the retaining catalytic mechanism (van Rantwijk et al. 1999). The first step of this mechanism is a nucleophilic attack on the anomeric carbon. The aglycone part of the sugar is released and a covalent intermediate is formed between the enzyme and the glycone. In the second step, the glycone serves as donor to either a water molecule (in hydrolysis) or to another acceptor such as a sugar (in transglycosylation).

Transglycosylation has so far been reported for GH5 β-mannanases in GH5_7 and GH5_10 (Couturier et al. 2013; Dilokpimol et al. 2011; Harjunpaa et al. 1999; Larsson et al. 2006; Schroder et al. 2006) as well as for one bacterial GH113 β-mannanase (Zhang et al. 2008). In the tomato plant, it has been suggested that a GH5_7 enzyme may take part in plant cell wall remodelling, due to displaying mannosyl transglycosylation/transferase activity in addition to β-mannanase activity (Schroder et al. 2006). At present, there are no reports on any native GH26 β-mannanase with the ability to perform transglycosylation. The molecular characteristics connected to transglycosylation capacity of β-mannanases are not completely revealed although some recent advances have been achieved (Hekmat et al. 2010; Dilokpimol et al. 2011; Rosengren et al. 2012). Interactions between the enzyme and its substrate take place at different subsites in the active site, where negative numbers represent glycone-binding subsites and positive numbers represent aglycone binding subsites (Davies et al. 1997). Strong aglycone binding has been suggested to be important for β-mannanases to be able to transglycosylate (Larsson et al. 2006; Rosengren et al. 2012). A previous study of the β-mannanase from *Trichoderma reesei*, TrMan5A, showed that engineering of the +2 subsite, mutating an Arg to a Lys, changed the substrate binding and significantly reduced transglycosylation onto sugars. Interestingly, although transglycosylation capacity with sugar acceptors was reduced, the ability to transfer to alcohol acceptors was retained (Rosengren et al. 2012). Besides *T. reesei*, other fungi such as for example *Aspergillus* sp. (Coutinho et al. 2009; Bien-Cuong et al. 2009) are biotechnologically interesting sources of β-mannanases and other carbohydrate converting enzymes. Three *Aspergillus nidulans* GH5 endo-β-1,4-mannanase genes (*manA*, *manB* and *manC*) were previously cloned into *Pichia pastoris* and the culture filtrate of induced cells showed hydrolytic activity on galactomannan (Bauer et al. 2006). The three *A. nidulans* β-mannanase sequences are all predicted to have a signal peptide for secretion. The gene products AnMan5A, AnMan5B and AnMan5C belong to GH5_7. However, they cluster in different groups in the phylogenetic tree of GH5_7 (Aspeborg et al. 2012). Biochemical characterisation of AnMan5A and AnMan5C has shown that they are both able to perform transglycosylation (Dilokpimol et al. 2011). The aim of this study was to characterise AnMan5B and by comparative studies with the paralogous AnMan5A and AnMan5C as well as with TrMan5A, which is the closest homolog with a solved 3D structure, further expand the knowledge about differences in transglycosylation, substrate binding and alcohol transfer by β-mannanases in GH5. This is interesting from a fundamental point of view and is also of great importance to improve the selection procedure of enzymes as tools for different biotechnological applications. Our findings reveal that although belonging to the same GH family and subfamily, the three paralogous *A. nidulans* β-mannanases and the homologous TrMan5A show significant differences in function, in respect to transglycosylation and acceptor usage.

## Materials and methods

### Sequence analysis

The amino acid sequence of *A. nidulans* AnMan5B (AN3297.2, UniProt entry Q5B833) was analysed for presence of signal peptide (SignalP server, http://www.cbs.dtu.dk/services/SignalP/), glycosylation sites (NetNGlyc 1.0 server, http://www.cbs.dtu.dk/services/NetNGlyc/), molecular weight and pI (Expasy ProtParam tool, http://web.expasy.org/protparam/). Multiple sequence alignments of GH5 β-mannanases were performed using Clustal Omega (http://www.ebi.ac.uk/Tools/msa/clustalo/), including characterised *Aspergillus* GH5 β-mannanases, structure determined GH5 β-mannanases and characterised fungal GH5 β-mannanases with more than 50 % sequence identity to AnMan5B. For accession entries, see Table 1.

**Table 1:** Amino acid sequences used in the multiple sequence alignment analysis of GH5 β-mannanases. Listed are: the sequence name displayed in the alignment, subfamily, organism and accession entries for UniProt (bold), PDB (underlined) or GeneBank (*italics*). Additional accession entries are shown in parenthesis

Name in alignment	Subfamily	Organism	Accession entry
AnMan5B	7	*Aspergillus nidulans*	**Q5B833**
TrMan5A_1QNR	7	*Trichoderma reesei*	1QNR (**Q99036**)
Phialophora	7	*Phialophora* sp.	**DSIF02**
Bispora	7	*Bispora* sp.	**B5LXD7**
A.niger_BK-01	7	*Aspergillus niger*	**B6V878**
A. aculeatus	7	*Aspergillus aculeatus*	**Q00012**
A. fumigatus	7	*Aspergillus fumigatus*	**Q4WBS1**
A. niger_CBS513.88	7	*Aspergillus niger*	**A2QKT4**
A. sulphureus	7	*Aspergillus sulphureus*	**Q2LE69**
PaMan5A_3ZIZ	7	*Podospora anserina*	3ZIZ (**B2B3C0**)
AnMan5A	7	*Aspergillus nidulans*	**Q5B7X2**
AnMan5C	7	*Aspergillus nidulans*	**Q5A253**
CsMan_4AWE	7	*Crysonila sitophila*	4AWE (*CCI55471.1*)
TpMan_3PZQ	7	*Thermotoga petrophila*	3PZQ (**A5IMX7**)
LeMan_1RH9	7	*Solanum lycopersicum*	1RH9 (**Q8L5J1**)
C.mixtus_1UUQ	7	*Celvibrio mixtus*	1UUQ (**Q6QT42**)
MeMan5A_2C0H	10	*Mytilus edulis*	2C0H (**Q8WPJ2**)
AkMan_3VUP	10	*Aplysia kurodai*	3VUP (*BAJ60954.1*)
TfMan_1BQC	8	*Thermobifida fusca* KW3	1BQC (**Q9ZF13**)
BaMan_3WHJ	8	*Bacillus agaradharens*	3WHJ (*AAN27517.1*)
B. sp N16-5_3JUG	8	*Bacillus* sp. N16-5	3JUG (**Q5YEX6**)
B. sp JAMB620_1WKY	8	*Bacillus* sp. JAMB-602	1WKY (**Q4W8M3**)

### Expression and purification of enzymes

*P. pastoris* X-33 strains harbouring genes encoding the full-length *A. nidulans* β-mannanases AnMan5A, AnMan5B or AnMan5C were obtained from Fungal Genetics Stock Center (FGSC, School of Biological Sciences, University of Missouri, Kansas City, MO) with accession no. 10088 (AN3358.2) for AnMan5A, no. 10086 (AN3297.2) for AnMan5B and no. 10106 (AN6427.2) for AnMan5C. The genes were previously cloned from cDNA of *A. nidulans* by others (Bauer et al. 2006). Enzymes were expressed and purified as in (Bauer et al. 2005) and following the recommendations in the Easy select *Pichia* expression kit manual (Invitrogen, Lidingö, Sweden). Cells were grown in 50 mL of buffered complex medium in a 250-mL flask in an orbital shaker (250 rpm) at 28 °C to an OD_600_ of 3–6. Aliquots of cells were then diluted to an OD_600_ of 1.0 with 200 mL of buffered complex medium containing methanol and incubated for 3–5 days with daily addition of methanol up to 0.5 %. The cultures were harvested by centrifugation (10 min, 5,000×*g*) and the supernatant was concentrated using an Amicon Ultra-15 centrifugal unit with 10 kDa cut-off (Millipore, Solna, Sweden). A 1-ml Ni-NTA Superflow cartridge (Qiagen, Germantown, USA) was used for purification of the His-tagged proteins according to the manufacturer’s recommendations. Protein purity was analysed with electrophoresis using NuPAGE Novex Bis-Tris gels 4–12 % (Invitrogen, Lidingö, Sweden) or 12 % polyacrylamide gels casted in lab. PageRuler™ Unstained Protein Ladder (Fermentas, Gothenburg, Sweden) was included for molecular weight estimation. Gels were stained with quick colloidal CBB-staining in water using microwave heating, as described in (Lawrence and Besir 2009). Purified enzymes were treated with endoglycosidase H (endoH) (New England BioLabs, Ipswich, MA) according to the manufacturer’s recommendations, in order to analyse N-glycosylation. Protein concentrations were determined using Pierce BCA Protein Assay (Thermo Fisher scientific, Rockford, USA) or NanoDrop® (ND-1000 spectrophotometer, Saveen Werner, Sweden) measuring absorbance at 280 nm and using extinction coefficients M^−1^ cm^−1^ [*ε*] = 90675 for AnMan5A, [*ε*] = 118510 for AnMan5B and [*ε*] = 115405 for AnMan5C, calculated from the amino acid sequences using the ExPASy tool ProtParam (http://web.expasy.org/protparam/). The identity of the proteins was confirmed with mass spectrometry and N-terminal sequencing of AnMan5B was done using a service provided by Proteome Factory (Proteome Factory AG, Berlin, Germany). The *Trichoderma reesei* β-mannanase TrMan5A was produced as in (Rosengren et al. 2012).

### Mannanase activity assay and enzyme characterisation

β-Mannanase activity was assayed with 0.5 % locust bean galactomannan (LBG) (Sigma St. Louis, MO) in 50 mM Na-citrate buffer using the 3,5-dinitrosalicylic acid (DNS) method as described previously (Stalbrand et al. 1993). Appropriately diluted enzyme (40 μl) was mixed with substrate (360 μl) and incubated for 10 min at 37 °C. The reaction was stopped by addition of DNS (600 μl), followed by 10 min boiling. After cooling the samples absorbance was measured at 540 nm and correlated to the amount of reducing ends based on a mannose standard curve. The pH-optimum was determined using 0.5 % LBG in 50 mM buffers of different pH (citrate pH 3–6, phosphate pH 6.5–8, citrate-phosphate pH 5.5–7 and Tris–HCl pH 7.5–9). The temperature optimum was determined by assaying the activity at optimal pH in temperatures from 35 to 70 °C as described above. The stability at 37 °C was tested by assaying the residual activity after incubating the enzymes for up to 24 h at pH 6.

### Kinetics with locust bean galactomannan

The kinetic constants for the LBG hydrolysis were determined by assaying the initial β-mannanase activity at different substrate concentrations (5 to 0.313 mg/mL) using the DNS assay as described above. The enzyme concentrations used were 145 nM AnMan5A, 1804 nM AnMan5B and 114 nM AnMan5C. The initial hydrolysis rate was plotted as a function of the substrate concentration by non-linear regression in a Michaelis-Menten graph to obtain the values for *k*_cat_, *K*_M_ and *k*_cat_/*K*_M_.

### Incubations with mannans and manno-oligosaccharides

Ivory nut mannan (5 mg/mL) (Megazyme, Bray, Ireland), mannobiose (M_2_) mannotriose (M_3_), mannotetraose (M_4_), mannopentaose (M_5_) and mannohexaose (M_6_) (Megazyme, Bray, Ireland) (5 mM) and manno-oligosaccharides with galactose substitutions; 6^1^-α-d-galactosyl-mannobiose (GM_2_), 6^1^-α-d-galactosyl-mannotriose (GM_3_), 6^3^-6^4^-α-d-galactosyl-mannopenatose (G_2_M_5_) (Megazyme, Bray, Ireland) (5 mM) were incubated overnight at 37 °C with AnMan5A (73 nM), AnMan5B (902 nM) and AnMan5C (57 nM). Na-citrate buffer 50 mM pH 5.5 was used with AnMan5A and AnMan5C and Na-citrate buffer 50 mM pH 6 was used with AnMan5B. The reactions were stopped by boiling and samples were analysed with thin-layer chromatography (TLC). Samples (2 μl) were applied onto a silica plate (Merck, Darmstadt, Germany) and the chromatography was run in a system of butanol/ethanol/water (10:8:7, *v*/*v*) for 8 h. The sugars were visualized using an *N*-(1-naphtyl) ethylenediamine dihydrochloride solution containing ethanol and sulfuric acid (Bounias 1980). The solution was poured on the TLC plate which was then dried and developed at 105 °C for 5–15 min. Manno-oligosaccharides M_1_–M_6_ (10 mM) were included as standards.

### Time course incubations with M_4_

A time course study of reactions with M_4_ was done by incubating 5 mM M_4_ with two different enzyme loads, based on LBG kinetics (0.05 and 1 nkat), of AnMan5A (15 and 290 nM), AnMan5B (450 and 9,045 nM) and AnMan5C (15 and 300 nM) for 0–24 h at 37 °C in 50 mM Na-citrate buffer pH 5.5 (AnMan5A and AnMan5C) or pH 6 (AnMan5B). Aliquots were withdrawn at different time points, the reactions were stopped by boiling and the samples were analysed with TLC as described above.

### Transglycosylation reactions with M_4_

Reactions with 30 mM M_4_ were set up with AnMan5B and TrMan5A (330 nM). Na-citrate buffer 50 mM pH 6 was used with AnMan5B and Na-acetate buffer 50 mM pH 4.5 was used with TrMan5A. Incubations were done at 37 °C for 0–180 min, aliquots were withdrawn at regular time intervals and the reactions were stopped by 5 min boiling. Analysis was done with high performance anion exchange chromatography with pulsed amperometric detection (HPAEC-PAD) and matrix-assisted laser desorption/ionisation time-of-flight mass spectrometry (MALDI-TOF MS). For HPAEC-PAD analysis a Dionex CX 500 system (Dionex, Sunnyvale CA, USA) including an AS50 auto sampler, a GP40 gradient pump and an ED40 electrochemical detector was used. Samples were run on CarboPac PA-100 analytical and guard columns and elution was performed with 100 mM NaOH at a flow rate of 1 ml/min. The injection volume was 10 μl and manno-oligosaccharides M_1_-M_6_ were used as standards. M_7_ products were quantified using the standard for M_6_. For MALDI-TOF MS analysis, samples (0.5 μl) were spotted directly onto a MALDI plate, supplied with matrix (10 mg/ml 2,5-dihydroxybenzoic acid (DHB) in water) and dried under warm air.

### Kinetics with manno-oligosacharides

The kinetic parameter *k*_cat_/*K*_M_ was determined with AnMan5B (2.5–100 nM) for M_3_-M_6_, by following the substrate conversion over time (0–180 min for M_3_ and 0–60 min for M_4_-M_6_) at low (≤50 μM) substrate concentration in 50 mM Na-citrate pH 6 at 37 °C. Duplicate samples were analysed with HPAEC-PAD using a CarboPac PA-100 column as described above. Ln(*S*_o_/*S*_t_) was plotted as a function of time (*t*) and *k*_cat_/*K*_M_ was calculated according to the Matsui equation; k = Ln(S_o_/S_t_), where *k* = ((*k*_cat_/*K*_M_)×[enzyme])×*t*, S_o_ = substrate concentration at time zero and *S*_*t*_ = substrate concentration at time *t* (Matsui et al. 1991). Data was analysed using GraphPadPrism software (Graph Pad Software Inc., San Diego, CA).

### Reactions with manno-oligosaccharides in H_2_^18^O

The relative preferred binding of manno-oligosaccharides was studied by incubations in H_2_^18^O followed by MALDI-TOF MS analysis, as previously described in (Hekmat et al. 2010; Rosengren et al. 2012). This approach is a development from experiments in a study of an endoglucanase where electrospray MS was used to analyse labelled cello-oligosaccharides (Schagerlof et al. 2009). In the present study, AnMan5A, AnMan5B and AnMan5C (31–34 nM) were incubated with 0.8 mM M_4_ or M_5_ in 93 % H_2_^18^O (Sigma Aldrich, Schnelldorf, Germany), 1 mM Na-citrate pH 5.5 (AnMan5A and AnMan5C) or pH 6 (AnMan5B) for 0–24 h at 8 °C. Incubations with ten times higher enzyme load (330 nM) of AnMan5B were also performed in the same way with 0.8 mM M_4_, M_5_ and M_6_. Samples (0.5 μl) were spotted directly onto a MALDI plate, supplied with matrix (10 mg/ml DHB in water) and dried under warm air.

### Product formation from M_4_ incubations in water-alcohol mixtures

For alcohol transfer reactions, 5 mM M_4_ were mixed with either 25 % (5.9 M) methanol or 25 % (2.6 M) butanol and incubated in 20 mM Na-citrate buffer pH 6 with AnMan5A, AnMan5B and AnMan5C (210–240 nM) for 0–24 h at 37 °C. The samples were boiled and diluted ten times in H_2_O before spotting them on a MALDI plate and applying matrix as described above.

### Transfer to G_2_M_5_ and ^18^O-labelled G_2_M_5_

To investigate the transfer capacity to G_2_M_5_, AnMan5B (240 nM) was incubated with 5 mM M_4_ and 5 mM G_2_M_5_ in 10 mM Na-citrate buffer pH 6 at 37 °C for 4 h. Reactions were also set up where G_2_M_5_ had been pre-labelled with ^18^O. The labelling was done by preparing 40 mM G_2_M_5_ in 97 % H_2_^18^O (Sigma Aldrich, Schnelldorf, Germany) and incubating at 90 °C for 30 min, which resulted in ~90 % ^18^O-labelling as deduced from the areas and intensities of the ^16^O- and ^18^O-labelled peaks in the MALDI-TOF MS analysis. AnMan5B (330 nM) was incubated with 0.8 mM M_4_ and 0.8 mM ^18^O-G_2_M_5_ in 1 mM Na-citrate buffer at 8 °C up to 2 h. The mixture contained 2 % H_2_^18^O that came from the ^18^O-G_2_M_5_ and therefore the incubations were done at 8 °C in order to avoid non-enzymatic incorporation of ^18^O. Samples (0.5 μl) were applied to the MALDI plate as described above.

### MALDI-TOF MS data acquisition and analysis

Acquisition and analysis of mass spectrometry data was done essentially as described in (Hekmat et al. 2010; Rosengren et al. 2012), using a MALDI-TOF 4700 Proteomics Analyzer (Applied Biosystems, Framingham, CA) in positive reflector mode for data acquisition. At a laser intensity of 5000, 50 sub spectra with 20 shots on each were accumulated from a sample spot to generate a spectrum. For data analysis, the Data Explorer version 4.5 software was used.

### Homology modelling

Based on the multiple sequence alignment, homology modelling of AnMan5B was performed with Prime (Schrödinger-LLC 2012), using the structure of the *Trichoderma reesei* β-mannanase TrMan5A as template (PDB entry 1QNR). N-/C-terminal end overhangs in the target sequence relative to the aligned template, were removed. Manual adjustments of model rotamers were made in Coot (Emsley and Cowtan 2004) to increase similarity to template. Model quality was evaluated with MolProbity (Chen et al. 2010), showing that 92.8 % of the amino acids were in favored Ramachandran regions and the model clashscore was 27.44 (Supplemental Table S1). Figures were prepared in PyMOL (Schrödinger-LLC 2013).

## Results

### Sequence analysis

**Fig. 1:**
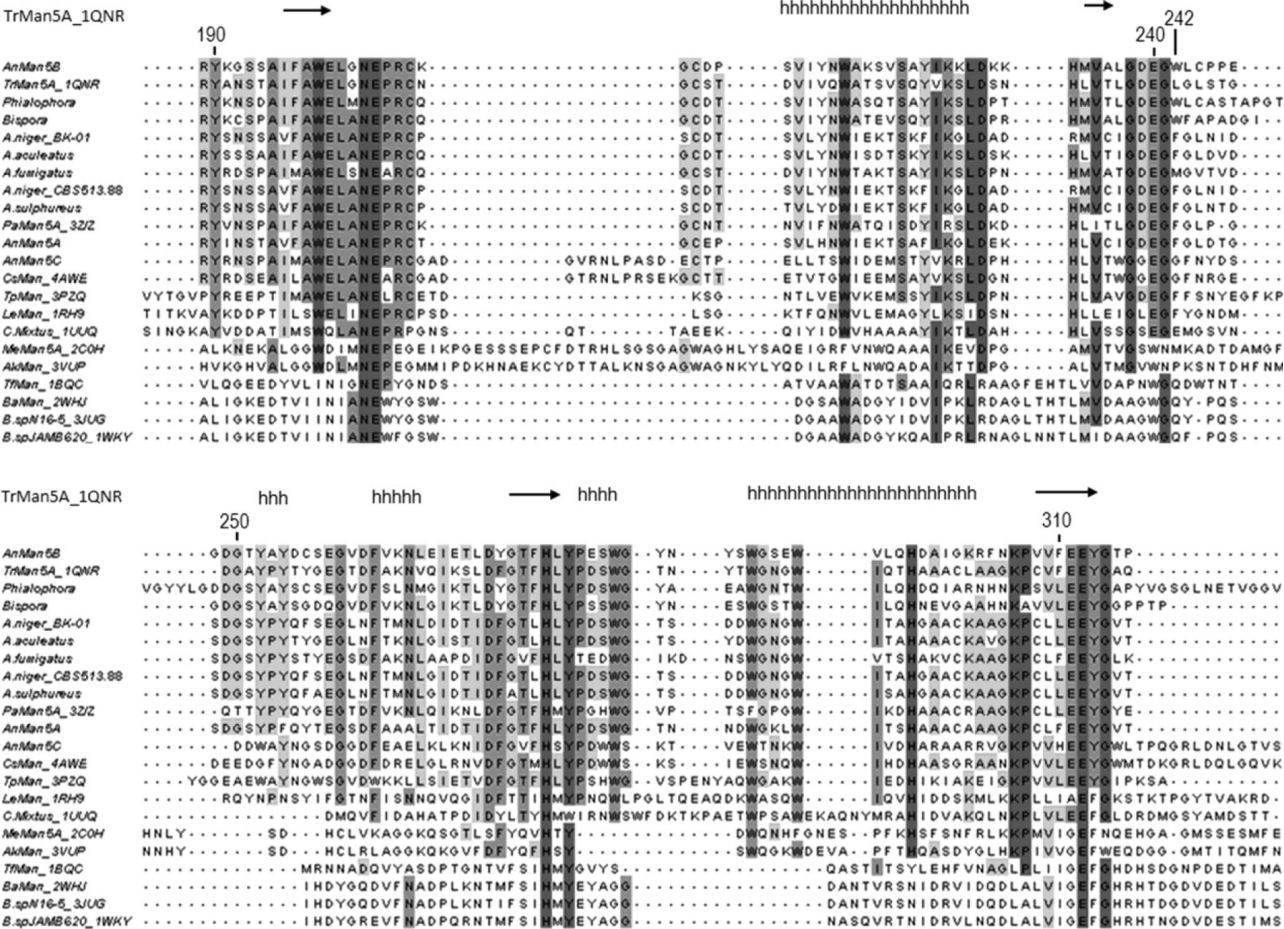
Multiple alignment of GH5 β-mannanases including (i) characterised *Aspergillus* β-mannanases (ii) structure determined GH5 β-mannanases (iii) characterised fungal β-mannanases with >50 % sequence identity to AnMan5B. The figure shows the region covering the two conserved catalytic residues E204 and E312, with numbering according to the AnMan5B sequence and with boxing in *grey* according to % sequence identity. The non-conserved Trp242 in AnMan5B is highlighted. The secondary structure of TrMan5A (from the PDB entry 1QNR) is shown as *arrows* for β-strands and *h* for helices. For accession entries, see Table 1

A multiple amino acid sequence alignment including AnMan5A, AnMan5B, AnMan5C, TrMan5A and characterised GH5 β-mannanases revealed at least seven amino acids that are highly conserved in GH5 (Hilge et al. 1998; Lo Leggio and Larsen 2002; Sabini et al. 2000; Sakon et al. 1996; Wang et al. 1993). For AnMan5B, these are R84, N203, E204, H277, Y279, E312 and W342, with E204 and E312 predicted to be the catalytic acid base and nucleophile respectively (Fig. 1). The arginine equivalent to R171 in the +2 subsite of TrMan5A (Sabini et al. 2000), that was previously shown to play role in the transglycosylation capacity of the enzyme (Rosengren et al. 2012), is also present in the *A. nidulans* enzymes as well as in the other subfamily 7 β-mannanases included in the sequence alignment.

### Expression, purification and basic characterisation

Active AnMan5A, AnMan5B and AnMan5C were expressed in *P. pastoris* and purified from the culture supernatant using metal ion chromatography, and the protein identities were confirmed by mass spectrometry. N-terminal sequencing of AnMan5B showed that the amino acid sequence started with APIGN, confirming signal peptide processing. AnMan5A and AnMan5C were previously shown to carry glycosylations (Dilokpimol et al. 2011). This was also the case with AnMan5B; the molecular weight decreased from 53 to 43 kDa after EndoH treatment (Supplemental Fig. S1a). AnMan5B has a pH-optimum of 6 (Supplemental Fig. S1b), comparable to AnMan5A and AnMan5C (Dilokpimol et al. 2011) and slightly higher than TrMan5A (Stalbrand et al. 1993). The temperature optimum of AnMan5B is 60 °C, also comparable to AnMan5A, AnMan5C (50 °C) (Dilokpimol et al. 2011) and TrMan5A (70 °C) (Stalbrand et al. 1993), and the enzyme remained stable when incubated 24 h at 37 °C and pH 6.

### Kinetics with LBG

Activity on galactomannans was previously reported for AnMan5A and AnMan5C, revealing a higher specific activity for AnMan5C (Dilokpimol et al. 2011). The Michaelis-Menten kinetic parameters with LBG determined for AnMan5A, AnMan5B and AnMan5C (Table 2) showed that AnMan5B has a lower *k*_cat_/*K*_M_ compared to AnMan5A and AnMan5C, and also compared to what was previously reported for TrMan5A (Rosengren et al. 2012). The low efficiency is mostly due to a low *k*_cat_, while *K*_M_ is comparable to AnMan5A and AnMan5C but higher compared to TrMan5A (Table 2).

**Table 2:** Kinetic parameters on LBG of AnMan5A, AnMan5B and AnMan5C and comparison to data reported for TrMan5A

Enzyme	*k*_cat_ (s^−1^)	*K*_M_ (mg.ml^−1^)	*k*_cat_/*K*_M_ (ml.mg^−1^.s^−1^)
AnMan5A	90	1.4	66.1
AnMan5B	15	2.6	5.9
AnMan5C	220	3.1	70.7
TrMan5A^a^	240	0.6	400

^a^Data from (Rosengren et al. 2012)

### End product formation from mannan and manno-oligosaccharides

When incubated over night with linear ivory nut mannan and manno-oligosaccharides M_3_-M_6_ AnMan5B generated M_2_ as end product in contrast to AnMan5A, AnMan5C and TrMan5A_,_ as analysed with TLC (data not shown). Under similar conditions, AnMan5A and TrMan5A produced M_2_ and M_3_ while AnMan5C produced M_1_ and M_2_. Similar to the other β-mannanases, mannobiose was not hydrolysed by AnMan5B.

### Product formation from incubations with M_4_

**Fig. 2:**
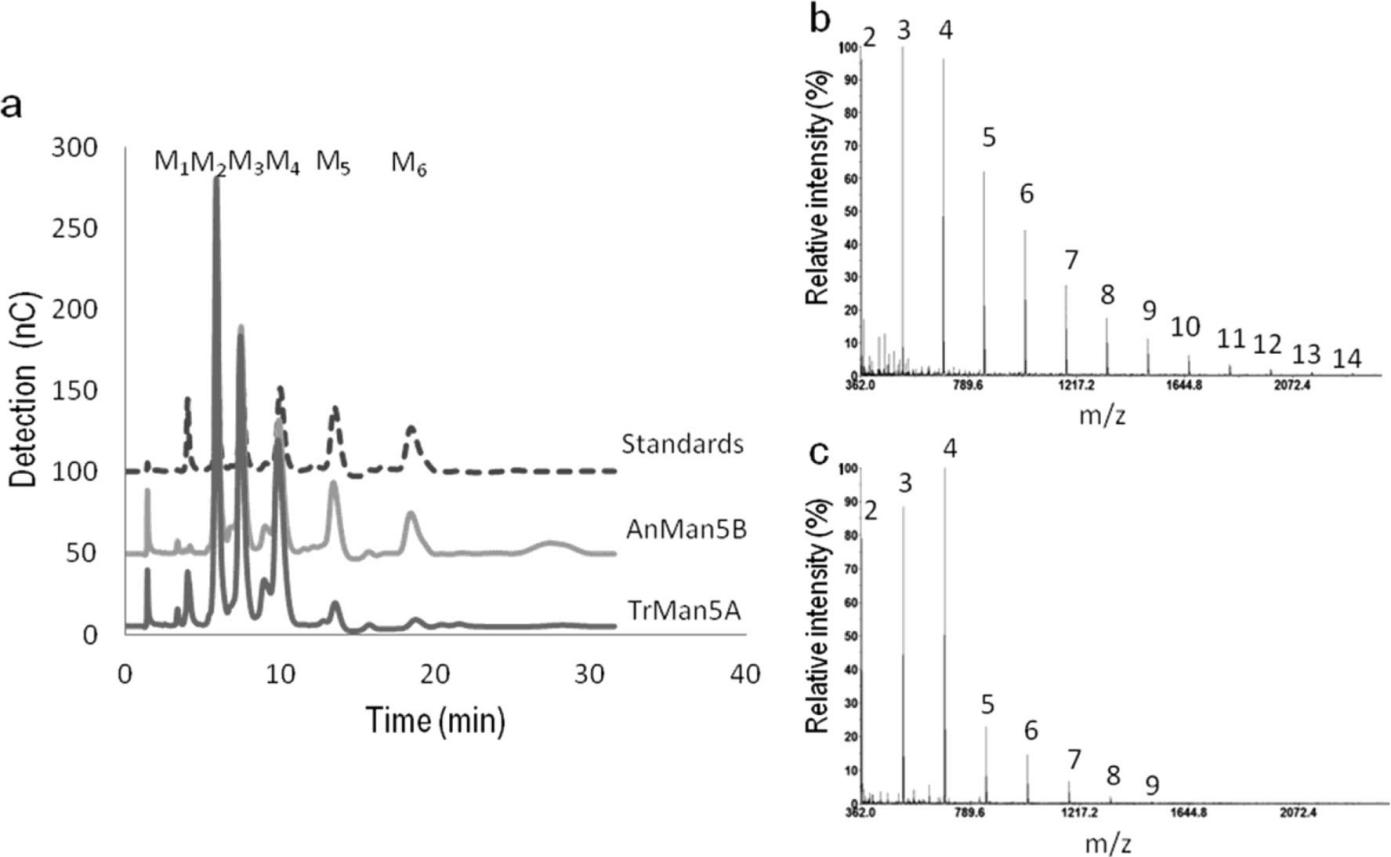
**a** HPAEC-PAD chromatogram of the 30 mM M_4_ incubation with AnMan5B and TrMan5A for 3 h. Manno-oligosaccharide standards are included and marked M_1_-M_6_. **b** MALDI-TOF MS analysis of the 3 h reaction with AnMan5B and M_4_, showing peaks of manno-oligosaccharides of DP2-DP14. **c** MALDI-TOF MS analysis of the 3 h reaction with TrMan5A and M_4_ showing peaks of manno-oligosaccharides of DP2-DP9

A time course analysis of the product formation from 5 mM M_4,_ analysed with TLC (Supplemental Fig. S2), showed that AnMan5B again stood out compared to AnMan5A, AnMan5C and TrMan5A. Similar to the other enzymes, AnMan5B produced M_3_ and M_2_ from M_4_, but in addition AnMan5B also appeared to generate transglycosylation products to a strikingly higher extent (Supplemental Fig. S2, lane B1). TrMan5A was previously shown to perform transglycosylation with M_4_ (Harjunpaa et al. 1999; Rosengren et al. 2012). AnMan5A and AnMan5C were also reported to generate transglycosylation products when incubated with high concentration (30 mM) of M_4_ (Dilokpimol et al. 2011). To further investigate and compare the transglycosylation capacity, AnMan5B and TrMan5A were incubated at similar conditions to those used previously with AnMan5A and AnMan5C (Dilokpimol et al. 2011). Using HPAEC-PAD analysis, transglycosylation products M_5_ and M_6_ were detected for both AnMan5B and TrMan5A, and in addition a M_7_ peak was seen in the chromatogram for AnMan5B (Fig. 2a). However, using MALDI-TOF MS analysis, products up to DP14 for AnMan5B (Fig. 2b) and DP9 for TrMan5A (Fig. 2c) were detected.

**Fig. 3:**
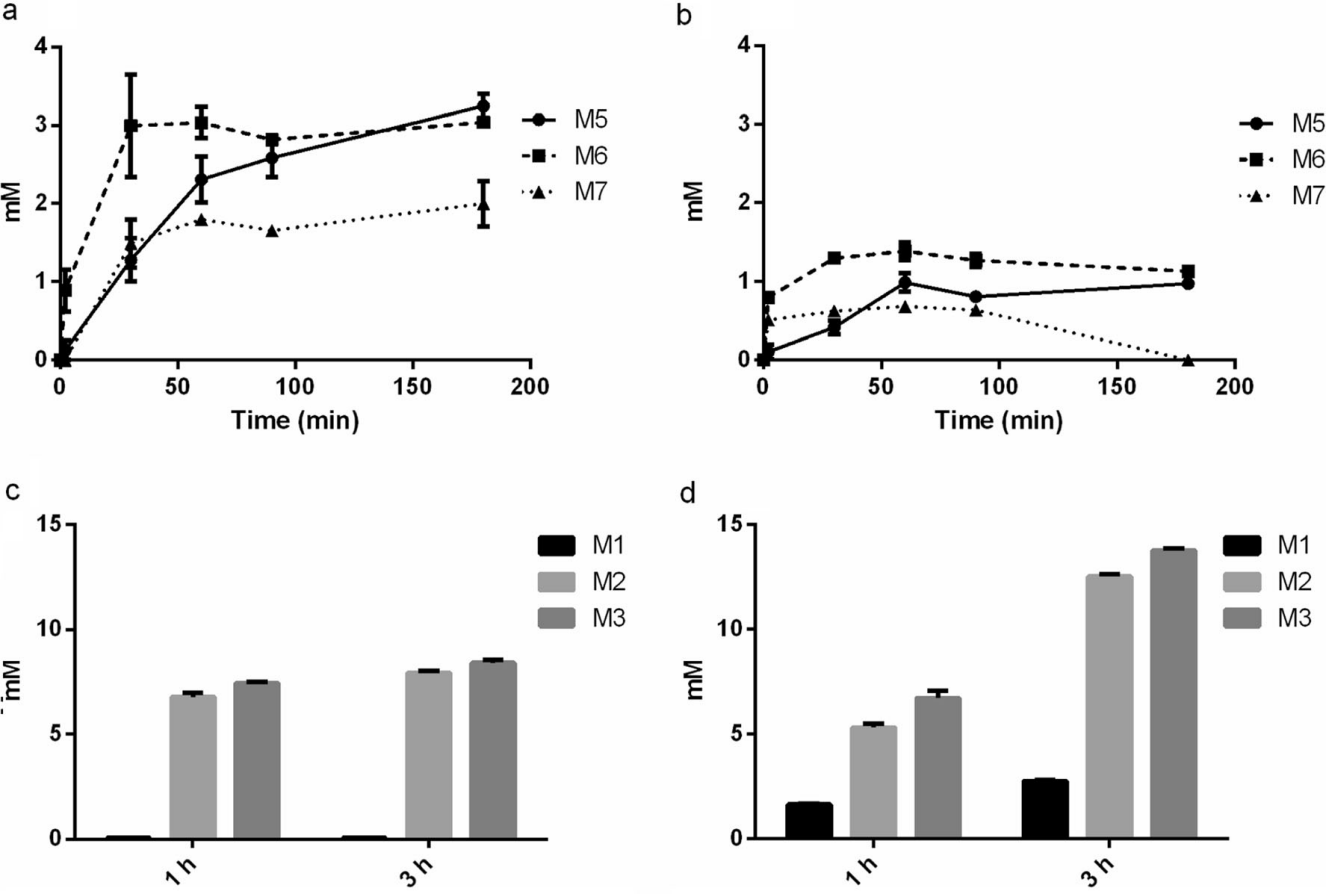
Products generated in reactions with 30 mM M_4_ using AnMan5B or TrMan5A, quantified with HPAEC-PAD. **a** Transglycosylation products M_5_-M_7_ generated with AnMan5B, **b** Transglycosylation products M_5_-M_7_ generated with TrMan5A, **c** Products M_1_-M_3_ generated with AnMan5B after 1 and 3 h, **d** Products M_1_-M_3_ generated with TrMan5A after 1 and 3 h. *Error bars* show deviation in double analysis

Quantification with HPAEC-PAD of the products generated in the 3 h time course reaction, showed that AnMan5B (Fig. 3a) generated higher amounts of transglycosylation products M_5_-M_7_ compared to TrMan5A (Fig. 3b). AnMan5B and TrMan5A differed not only in the amount of transglycosylation products generated at comparable substrate conversions between 40 and 80 %, but also in the product profile. AnMan5B continuously generated more of the transglycosylation products M_5_-M_7_ (Fig. 3a) while keeping the amount of M_2_ and M_3_ products rather constant (Fig. 3c). With TrMan5A, the transglycosylation products decreased after a certain time (60 min) of incubation (Fig. 3b) and the amount of M_1_-M_3_ increased (Fig. 3d).

Overall, AnMan5B differed in product formation compared to TrMan5A, and also compared to AnMan5A and AnMan5C (Dilokpimol et al. 2011), when incubated with a high concentration of M_4_ (30 mM). AnMan5B gave a higher total yield of transglycosylation products M_5_-M_7_ compared to the other three enzymes. AnMan5C generated more M_5_ and M_6_ than both AnMan5A and TrMan5A. In comparison, AnMan5B generated similar amounts of M_6_ as AnMan5C, but higher amounts of M_5_ and also substantial amounts of M_7_, not seen for AnMan5C (Dilokpimol et al. 2011). These results together with the detection of products up to DP14 in the MALDI-TOF MS analysis (Fig. 2b) and the absence of mannose production while M_3_ is produced (Figs. 2a and 3c) indicate an exceptionally efficient glycosyl transfer capacity by AnMan5B.

### Kinetics with manno-oligosaccharides

To get further insight in interactions and catalytic efficiency with shorter substrates, *k*_cat_/*K*_M_ on manno-oligosaccharides was determined for AnMan5B. A low substrate concentration (50 μM) was used, to limit transglycosylation. The threshold in catalytic efficiency was between M_3_ and M_4_. AnMan5B had a higher *k*_cat_/*K*_M_ on M_4_ compared to AnMan5A, AnMan5C and TrMan5A (Table 3). The similar *k*_cat_/*K*_M_ on M_4_ and M_5_ for AnMan5B indicated that occupation of substrate at additional subsites above four did not considerably enhance the catalytic efficiency, although a slight increase was seen with M_6_. Thus, this suggests that occupation of at least four subsites is required for efficient catalysis. This distinguishes AnMan5B from what was reported for AnMan5A, AnMan5C and TrMan5A, where *k*_cat_/*K*_M_ constantly increased with increasing DP of the manno-oligosaccharides up to the tested M_6_ (Table 3).

**Table 3:** Kinetic efficiency on manno-oligosaccharides for AnMan5B and comparison to data reported for AnMan5A, AnMan5C and TrMan5A

Substrate	*k*_cat_/*K*_M_ (s^−1^ mM^−1^)			
	AnMan5B	AnMan5A^a^	AnMan5C^a^	TrMan5A^b^
M_3_	0.16 ± 0.02	Not reported	Not reported	Not reported
M_4_	40 ± 4.3	6	7	19.4
M_5_	36.6 ± 2.6	23	61	163
M_6_	61.6 ± 6.7	109	215	400

^a^Data from Dilokpimol et al. (2011)

^b^Data from Rosengren et al. (2012)

### Incubations with manno-oligosaccharides in H_2_^18^O

Some information on preferred substrate binding was previously reported for AnMan5A, AnMan5C (Dilokpimol et al. 2011) and TrMan5A (Rosengren et al. 2012). To get more detailed information on how manno-oligosaccharides bind to the active site of AnMan5B, incubations were set up in ^18^O-labelled water as described by (Couturier et al. 2013; Hekmat et al. 2010; Rosengren et al. 2012) to analyse the cleavage pattern. Non-enzymatic incorporation of ^18^O was prevented by setting up the incubations at low temperature (8 °C), in accordance with our previous study (Hekmat et al. 2010). Interestingly, at the same conditions as used with AnMan5A and AnMan5C (data not shown) and previously with other β-mannanases (Couturier et al. 2013; Hekmat et al. 2010; Rosengren et al. 2012), no ^18^O-labelled products could be detected with AnMan5B in initial stages (up to 1 h) of substrate conversion. In addition, transglycosylation products up to DP9 from M_4_ and DP12 from M_5_ and M_6_ were detected in the MALDI-TOF MS spectra (Supplemental Fig. S3a, b and c respectively), which was not seen with any of the other enzymes tested using this relatively low substrate concentration (0.8 mM).

No ^18^O-labelled product could be seen with AnMan5B until the reaction was allowed to proceed substantially further (up to 24 h incubation), which resulted in detection of small peaks of ^18^O-labelled M_2_ and M_3_ in the MALDI-TOF MS spectra (data not shown). The absence of ^18^O-labelled products, together with the substantial amount of transglycosylation products detected in the same reactions, strongly indicates that saccharides rather than water acted as main acceptor for AnMan5B. Thus, AnMan5B has exceptionally efficient glycosyl transfer capacity also at relatively low substrate concentrations (0.8 mM). The non-labelled (^16^O) products with lower DP than the original substrate (for example M_2_ and M_3_ from M_4_) are then produced in the initial aglycone release, at the first step of the reaction where the covalent intermediate between enzyme and substrate is formed.

### Transfer to alcohol acceptors

**Fig. 4:**
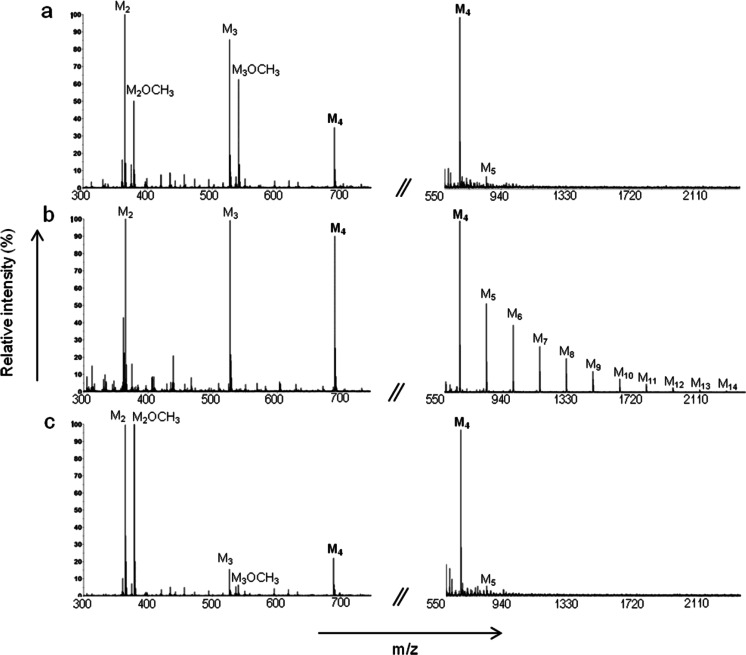
MALDI-TOF MS analysis of reactions with M_4_ and MeOH. The spectra show samples incubated at the same conditions for 1,5 h with **a** AnMan5A, **b** AnMan5B and **c** AnMan5C

The interesting finding that AnMan5B seemed to prefer saccharides as acceptor molecules instead of water, at tested substrate concentrations ≥0.8 mM, lead us to analyse whether alcohols could be used as acceptors. Transfer to methanol (Fig. 4) and butanol (data not shown) was tested with AnMan5B and compared to AnMan5A and AnMan5C, using 5 mM M_4_ as donor substrate, in a similar way to what was done previously with TrMan5A (Rosengren et al. 2012). MALDI-TOF MS analysis showed that AnMan5A gave alcohol conjugates with M_2_ and M_3_ (Fig. 4a), similarly to TrMan5A (Rosengren et al. 2012). AnMan5C generated alcohol conjugates with M_2_ to a higher extent (deduced from the relative peak intensities) compared to the other enzymes, and gave only little amount of M_3_-conjugate (Fig. 4c). However, when AnMan5B was incubated under similar conditions to AnMan5A and AnMan5C, no detectable alcohol conjugates were generated and only negligible amounts were detected even after prolonged incubation time (>5 h, data not shown). On the other hand, substantial amounts of transglycosylation products up to DP14 were detected in the spectra for AnMan5B (Fig. 4b) but not for AnMan5A (Fig. 4a) or AnMan5C (Fig. 4c).

### Transfer to G_2_M_5_ by AnMan5B

**Fig. 5:**
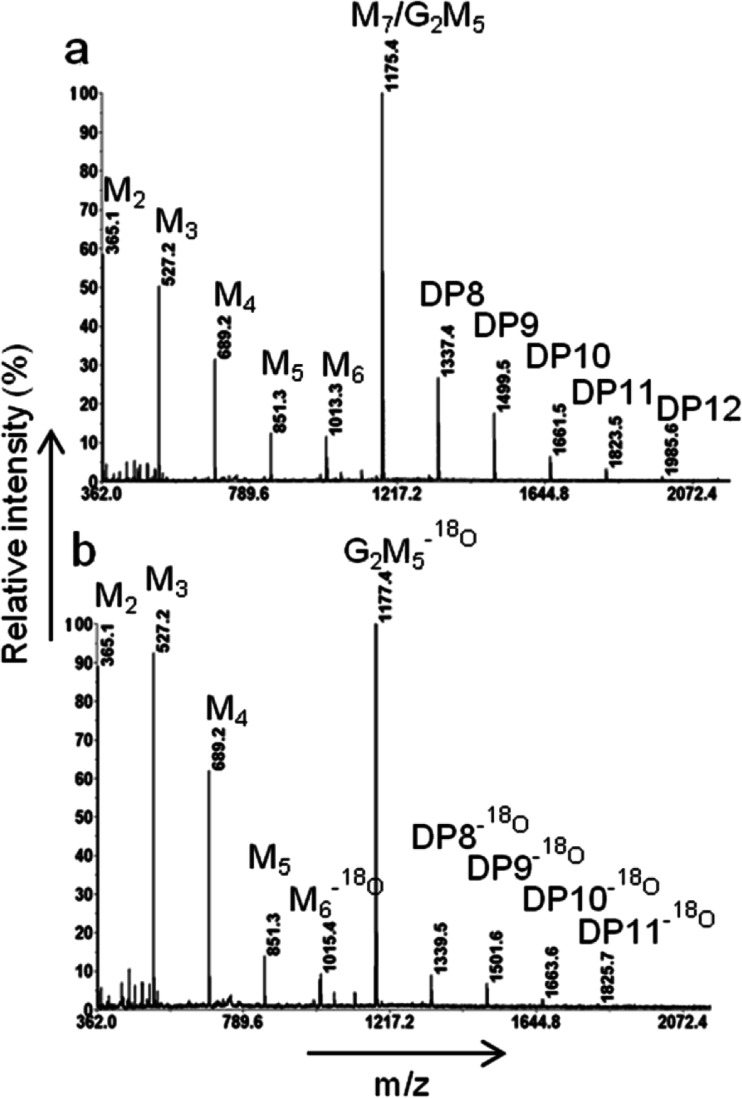
MALDI-TOF MS spectra showing the transfer to G_2_M_5_ by AnMan5B using M_4_ as donor substrate. **a** Spectra from the incubation with 5 mM M_4_ and 5 mM G_2_M_5_, giving transfer products of DP8-DP12. **b** Spectra from the incubation with 0.8 mM M_4_ and 0.8 mM G_2_M_5_-^18^O giving transfer products of DP8-DP11 with additional mass of 2 Da, confirming that there is transfer to the ^18^O-labelled G_2_M_5_. The ^18^O-labelled M_6_ is a contaminant in the G_2_M_5_-^18^O preparation

The results from the alcohol transfer reactions indicated that AnMan5B was less efficient in transferring to alcohol acceptors compared to AnMan5A, AnMan5C and TrMan5A, while the transfer to manno-oligosaccharides was considerably more efficient. Mannopentaose substituted with galactose at positions two and three, G_2_M_5_, was not hydrolysed by AnMan5B (data not shown). Interestingly, MALDI-TOF MS analysis showed that AnMan5B seemed to be able to use G_2_M_5_ as acceptor, using M_4_ as donor substrate, generating conjugates from DP8 to DP12 (Fig. 5a). To distinguish transfer products from linear manno-oligosaccharides with the same DP and therefore the same molecular weight, incubations where G_2_M_5_ had been pre-labelled with ^18^O were set up. The detection of DP8-DP11 products with additional masses of 2 Da (resulting from the ^18^O instead of ^16^O on the G_2_M_5_) confirmed that AnMan5B indeed used G_2_M_5_ as acceptor (Fig. 5b).

### Homology modelling

**Fig. 6:**
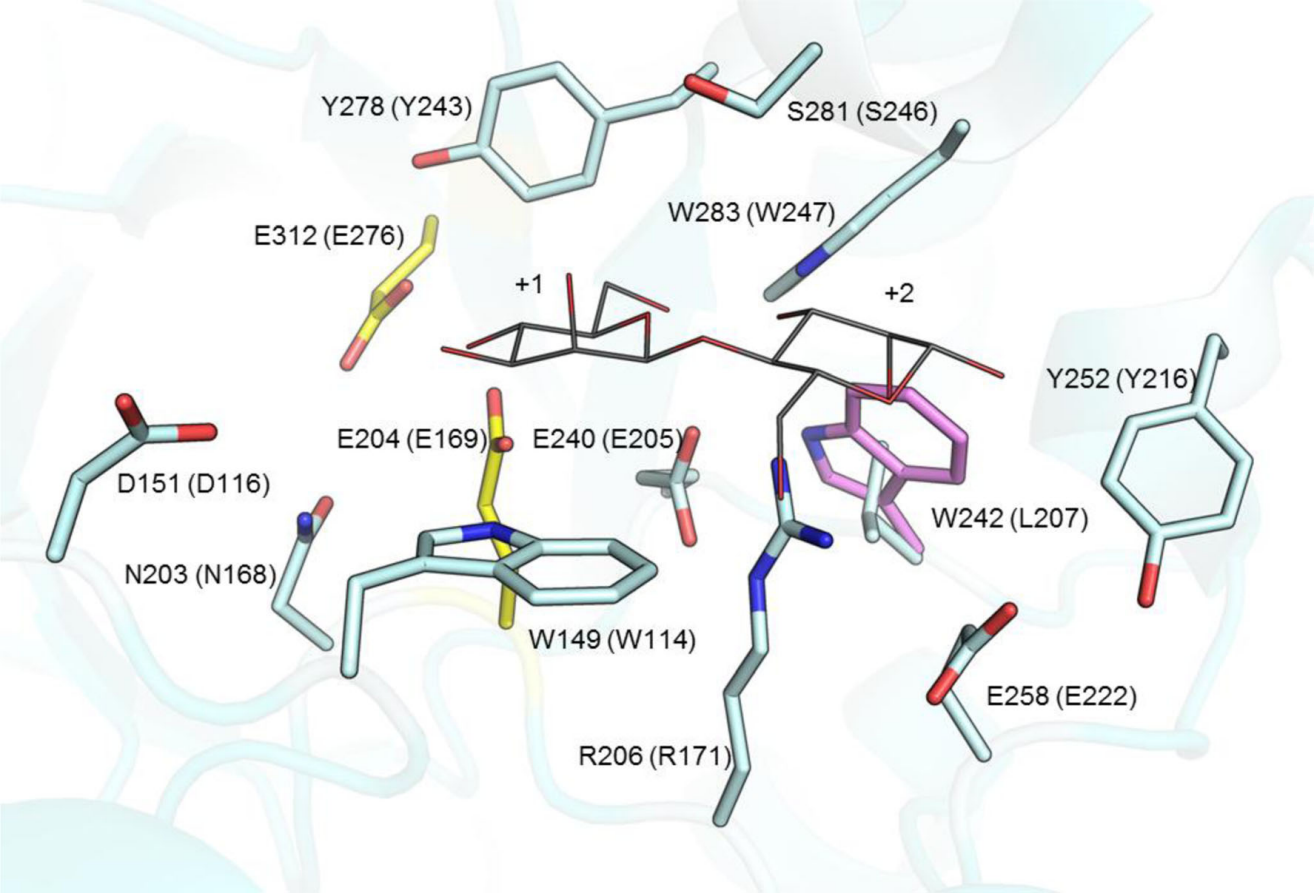
Close up on the active site showing an overlay of the structure of TrMan5A (*cyan*) in complex with mannobiose (*grey*) at subsite +1 and +2 and the homology model of AnMan5B (*violet*). Oxygens are in *red*, nitrogens are in *blue* and the catalytic residues are shown in *yellow*. The amino acids are numbered according to the sequence of AnMan5B with corresponding number in the 1QNR template in *parenthesis*. Residues within 6 Å of the mannobiose are conserved between the model and the template, except for W242 in the AnMan5B, which corresponds to L207 in TrMan5A

As an initial step towards finding structural features that are responsible for the functional properties of AnMan5B, a homology model was generated using the crystal structure of the catalytic domain of β-mannanase TrMan5A as template (PDB entry 1QNR (Sabini et al. 2000), 48 % sequence identity to AnMan5B). Compared to the template the AnMan5B model showed an overall well-conserved catalytic site with no apparent differences in subsites −2 and −1 (data not shown). One notable difference could be seen at a non-conserved position on the aglycone side (Fig. 6), where AnMan5B has Trp242 instead of Leu207 in the template. Trp242 could potentially contribute to sugar monomer binding in subsite +2.

## Discussion

Fundamental studies of β-mannanases and other enzymes which are active on plant cell wall carbohydrates not only increases our knowledge about natural biomass conversion, but also provides important information that can guide in the selection of enzymes for certain applications. Sequence analysis and classification of β-mannanases into families and subfamilies provide useful information, but cannot alone predict the fine-tuned specificity of individual enzymes. The functional diversity needs to be further investigated by detailed biochemical characterisation, as exemplified in the present study where paralogous and homologous β-mannanases belonging to the same family, and even the same subfamily (GH5_7), were shown to have quite different functional properties. In terms of hydrolase/transglycosylase activity, plant enzymes acting on xyloglucan are among the most well studied (Eklof and Brumer 2010; Eklof et al. 2013; Kaewthai et al. 2013) and only a few plant β-mannanases have been studied (Schroder et al. 2006; Wang et al. 2014). In this study, we focus on differences in transglycosylation capacity among fungal *A. nidulans* β-mannanases and related enzymes of GH5_7. Transglycosylation is of interest, e.g. in enzyme catalysed synthesis, but could potentially be counterproductive for efficient mannan degradation. Differences were seen in hydrolytic efficiency on LBG, product profiles, transglycosylation capacity with manno-oligosaccharides, and in acceptor usage.

A β-mannanase such as AnMan5B, with low *k*_cat_/*K*_M_ on galactomannan (Table 2) may be specialized to have other functions than hydrolysing polymeric substrates. The low hydrolytic efficiency on LBG, together with the absence of ^18^O-labelled products when incubated with manno-oligosaccharides in H_2_^18^O (Supplemental Fig. S3), strongly suggests that AnMan5B prefers to use sugars as acceptors instead of water. The yield of transglycosylation products from mannotetraose (Fig. 3a) and the MALDI-TOF MS detection of products up to DP14 (Fig. 2b) showed that AnMan5B indeed has an exceptionally good capacity to generate transglycosylation products. The amount of M_2_ and M_3_ remained rather constant during the time course studied (Fig. 3c) and no mannose was detected although M_3_ was produced (Fig. 2a). This further indicates that the main catalytic event for AnMan5B is transglycosylation.

Increasing the substrate concentration increases the possibility of formation of transglycosylation products (Dias et al. 2004; Sinnott 1990). Interestingly, with AnMan5B, transglycosylation products were generated even at relatively low substrate concentrations (Supplemental Fig. S3), highlighting its potential in sugar synthesis applications. A great advantage of native transglycosylation as seen with AnMan5B is that natural sugars can be used in the synthesis, compared to the glycosynthase approach where fluorinated substrates are required (Hancock et al. 2006). Furthermore, the capability of AnMan5B to transfer to G_2_M_5_ (Fig. 5) demonstrates the synthesis of substituted complex products of defined size, which is otherwise difficult to achieve.

AnMan5B showed a significantly lower capacity to transfer to alcohol acceptors compared to AnMan5A and AnMan5C (Fig. 4). In the case of methanol, this is potentially connected to the preference for using sugars as acceptors instead of water, as lower alcohols could be regarded as water analogues. Exclusion of water has previously been reported as one factor responsible for high sugar synthesis capacity by a GH31 glycosidase (Larsbrink et al. 2012). AnMan5A and AnMan5C were both reported to perform transglycosylation reactions with mannotetraose (Dilokpimol et al. 2011), but to a lower extent than AnMan5B. In addition, they were both more efficient in LBG hydrolysis and both AnMan5A and AnMan5C generated hydrolysis products with manno-oligosaccharides when incubated in H_2_^18^O, similar to TrMan5A (Rosengren et al. 2012). In the reactions with alcohols, AnMan5A and AnMan5C both produced conjugates with methanol and butanol, which was not seen with AnMan5B. The product distribution of alcohol conjugates differed, reflecting the preferred binding of mannotetraose. AnMan5C generated almost exclusively alcohol conjugate with M_2_ while AnMan5A generated conjugates also with M_3_ (Fig. 4). TrMan5A was also reported to produce M_2_- and M_3_-conjugates with methanol and butanol (Rosengren et al. 2012). Interestingly, a subsite +2 mutant of TrMan5A with impaired transglycosylation capacity to sugars was able to transfer to alcohols, indicating that transglycosylation capability onto sugar acceptors is not necessarily a criterion for alcohol transfer capability.

Kinetic analysis with manno-oligosaccharides showed that AnMan5B requires occupation of at least four subsites for efficient substrate conversion (Table 3). From the incubations in H_2_^18^O, although no ^18^O-labelled products were generated, the product profile could reveal the preferred binding of substrate. AnMan5B prefers to bind substrate so that it occupies at least subsite −2 and +2. The initial product formation from M_4_ was M_2_ and M_6_, where non-labelled M_2_ was produced in the aglycone release step and M_6_ was produced by transglycosylation onto another M_4_ acceptor molecule. When reacting with M_5_, the first aglycone product released was non-labelled M_3_ and the transglycosylation product was M_7_, indicating binding from subsite −2 to +3. The same pattern was seen in the transfer reactions with G_2_M_5_, where the initial products were M_2_ and G_2_M_7_ (data not shown).

Structural data can provide insight in molecular details that are connected to certain functions. Of the six crystal structures of eukaryotic GH5 β-mannanases present to date (Bourgault et al. 2005; Couturier et al. 2013; Goncalves et al. 2012; Larsson et al. 2006; Mizutani et al. 2012; Sabini et al. 2000), transglycosylation has been reported for four of the enzymes. Three are in GH5_7 (Couturier et al. 2013; Sabini et al. 2000; Schroder et al. 2006) and one is in GH5_10 (Larsson et al. 2006). The three β-mannanase 3D structures from subfamily GH5_7 all have an arginine in the +2 subsite and the one in subfamily GH5_10 has two tryptophans in the +2 subsite. The arginine that provides mannosyl affinity in +2 in TrMan5A, shown to be important for transglycosylation capacity (Rosengren et al. 2012), is also present in AnMan5A, AnMan5B and AnMan5C. In addition, it has previously been shown that a non-conserved tryptophan located in subsite +1 of AnMan5C was responsible for the higher yield of transglycosylation products generated with AnMan5C compared to AnMan5A (Dilokpimol et al. 2011). The importance of subsites in the aglycone region for efficient transglycolsylation has also been shown for GH18 chitinases and a GH10 xylanase (Armand et al. 2001; Zakariassen et al. 2011).

An initial step in the direction of pinpointing structural features that are connected to specific functions was taken with AnMan5B by creating a homology model. The model showed an overall well-conserved catalytic site compared to the template structure TrMan5A, with the only notable difference seen on the aglycone side where AnMan5B had a tryptophan (Trp242) instead of a leucine in the template (Fig. 6). Tryptophan is known to be involved in stacking interactions with sugar in the active site of carbohydrate active enzymes (Eide et al. 2013; Larsson et al. 2006). Most likely, there is a combination of factors in the structure of AnMan5B that contribute to high transglycosylation capacity, where R206, analogous to R171 in TrMan5A, might play one of the roles in providing affinity in the aglycone subsites, potentially together with W242.

This study highlights the importance of the selection of a suitable β-mannanase depending on the application. We have provided insight in features that play a role in different functions of β-mannanases. In this respect, AnMan5B is clearly different from its paralogs AnMan5A and AnMan5C and the homolog TrMan5A. All these enzymes, and several other β-mannanases of GH5, can transglycosylate. This is useful for enzyme catalysed synthesis. However, from lower manno-oligosacharides AnMan5B is producing the longest transglycosylation products so far detected (DP14). AnMan5B has also a significantly lower *k*_cat_ and *k*_cat_/*K*_M_ using polymeric mannan (LBG) as a substrate in an assay detecting hydrolytic activity. Furthermore, the absence of ^18^O-labelled products from manno-oligosaccharide incubations in H_2_^18^O and the build-up of significant amounts of long transglycosylation products suggest that AnMan5B is prone to use saccharide acceptors rather than water. This view is further underlined by AnMan5B not being able to use methanol as acceptor, given the structural similarity to water. A contributing factor to these observations may be high affinity for saccharide acceptors binding in the aglycone region of AnMan5B as indicated by the presence of Trp242 potentially contributing to sugar binding in the +2 subsite, in addition to Arg206 which is semi-conserved in GH5 and shown to be important for transglycosylation (Rosengren et al. 2012).

## Electronic supplementary material

ESM 1 (PDF 530 kb)
