# Supplementary material for: An Aspergillus nidulans β-mannanase with high transglycosylation capacity revealed through comparative studies within glycosidase family 5
Source: Appl Microbiol Biotechnol. 2014 Jun 21;98(24):10091–104. doi: 10.1007/s00253-014-5871-8 (PMC4237917; doi:10.1007/s00253-014-5871-8)
Supplement: Supplementary file 1 — (PDF 530 kb) [file 253_2014_5871_MOESM1_ESM.pdf]

**Electronic supplementary material**
**Applied Microbiology and Biotechnology**

# An *Aspergillus nidulans* β-mannanase with high transglycosylation capacity revealed through comparative studies within glycosidase family 5

Anna Rosengren[1], Sumitha K. Reddy[1], Johan Svantesson Sjöberg[1], Oskar Aurelius[1], Derek T. Logan[1], Katarína Kolenová[1,2] and Henrik Stålbrand[1#]

[1]Department of Biochemistry and Structural Biology, Lund University,
P.O. Box 124, S-221 00, Lund, Sweden

[2]Present address: Institute of Chemistry, Center for Glycomics, Slovak Academy of Sciences, Dúbravská cesta 9, 845 38 Bratislava, Slovakia

[#]To whom correspondence should be addressed:
Henrik.Stalbrand@biochemistry.lu.se, Tel: +46-46-222 8202, Fax +46-46-2224116

**Supplemental Table S1** Quality parameters of the AnMan5B homology model, evaluated with MolProbity. For comparison the values for the template are shown in parenthesis.

| | AnMan5B model (template, 1QNR) |
|---|---|
| Number of residues | 350 (344) |
| Sequence identity | 48 % (-) |
| Ramachandran favored | 92.82 % (97.37 %) |
| Ramachandran outliers | 1.72 % (0.00 %) |
| Poor rotamers | 5.72 % (0.71 %) |
| Clashscore | 27.44 (4.53) |
| Residues with bad bonds | 0.29 % (0.29 %) |
| Residues with bad angles | 2.86 % (1.74 %) |

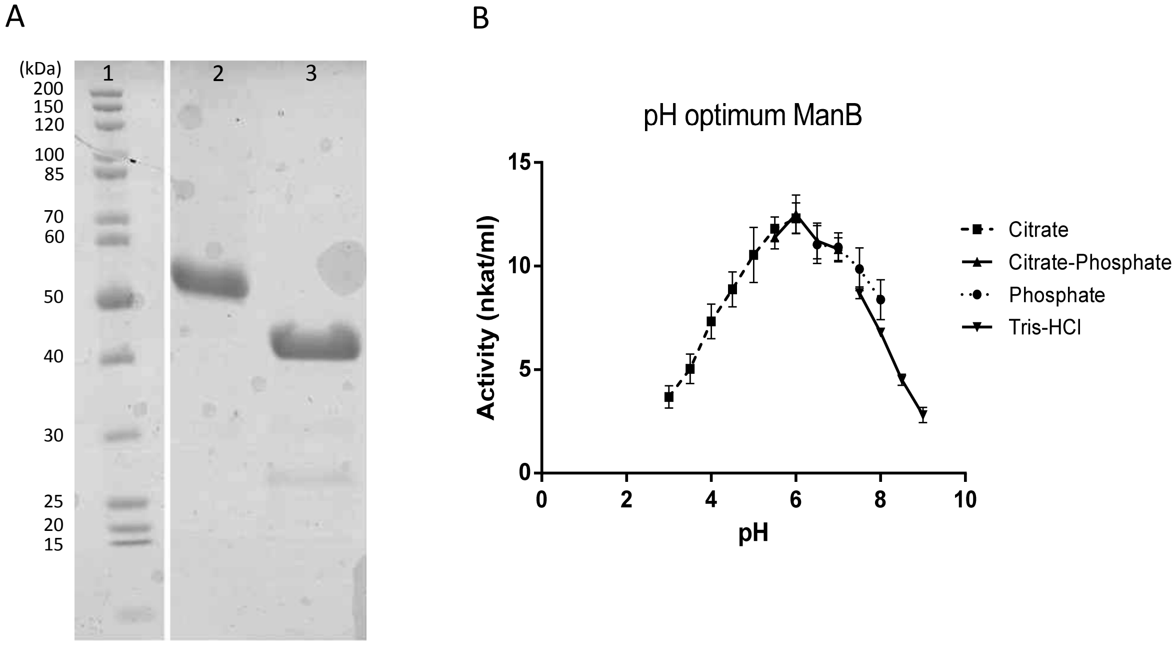


**Supplemental Fig. S1 a**; SDS-PAGE from the deglycosylation study showing molecular weight marker (1), purified AnMan5B (2) and purified AnMan5B treated with endoH (3). **b**; pH optimum for AnMan5B, using LBG as substrate.

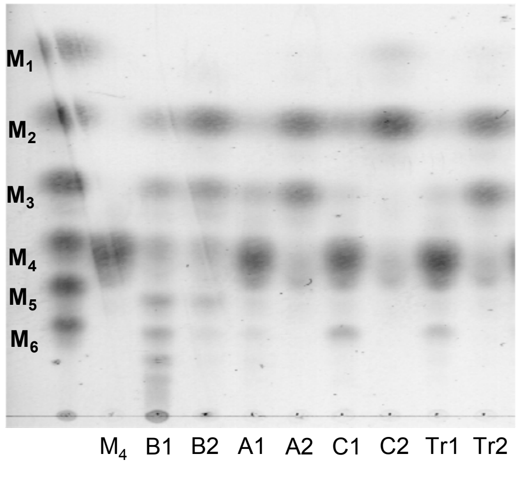


**Supplemental Fig. S2** TLC analysis of 5 mM $M_4$ incubation for 0.5 h, using two different enzyme loads, based on activity on LBG. AnMan5B; B1: 450 nM and B2: 9045 nM, AnMan5A; A1: 15 nM and A2 290 nM, AnMan5C; C1: 15 nM and C2: 300 nM and TrMan5A; Tr1: 9 nM and Tr2: 190 nM. Standard manno-oligosaccharides $M_1$-$M_6$ are shown to the left.

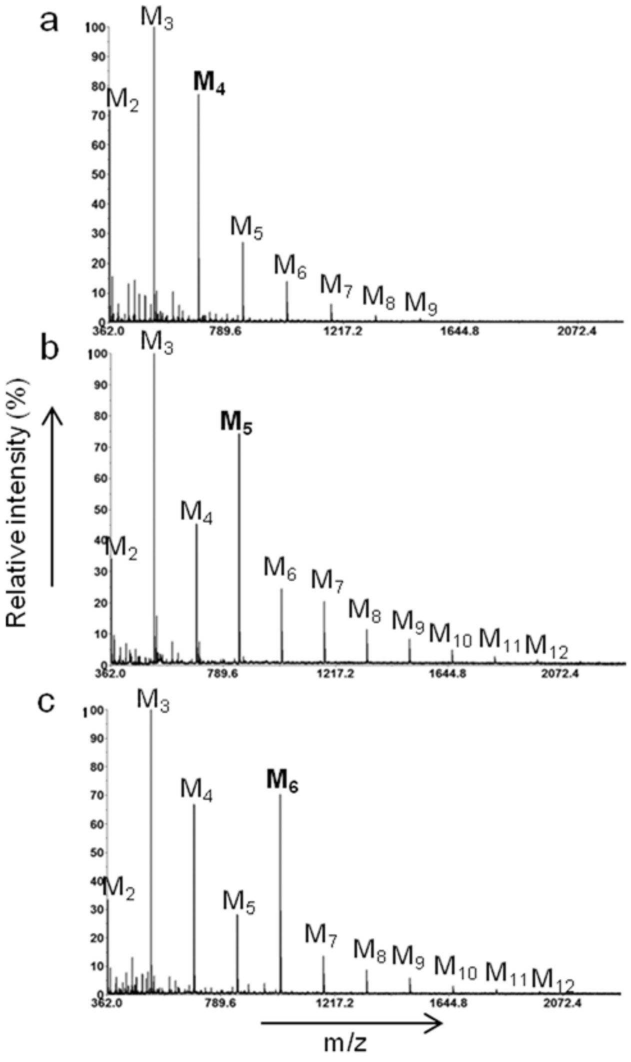


**Supplemental Fig. S3** MALDI-TOF MS spectra of AnMan5B incubations in $H_2{}^{18}O$ with **a**; $M_4$ 60 min, **b**; $M_5$ 60 min and **c**; $M_6$ 30 min. Products with lower DP that the substrates are present in the spectra, as well as transglycosylation products up to DP9 (a) and DP12 (b, c). No $^{18}O$-labeled oligosaccharide was detected.
